# Antihypertensive medicine use differs between Ghana and Nigeria

**DOI:** 10.1186/s12872-022-02799-z

**Published:** 2022-08-10

**Authors:** Samantha A. Hollingworth, Daniel Ankrah, Benjamin S. C. Uzochukwu, Chinyere C. Okeke, Francis Ruiz, Emily Thacher

**Affiliations:** 1 School of Pharmacy, The University of Queensland, 20 Cornwall St, Woolloongabba, QLD 4102 Australia; 2 Faculty of Pharmacy and Pharmaceutical Sciences, Kwame Nkrumah University of Science and Technology, Kumasi, Ghana; 3 Department of Pharmacy, Korle Bu Teaching Hospital, Accra, Ghana; 4 Department of Community Medicine, College of Medicine, University of Nigeria, Enugu, Nigeria; 5 International Decision Support Initiative, London School of Hygiene and Tropical Medicine, London, UK; 6 Seattle, USA

**Keywords:** Antihypertensive medicines, Hypertension, Ghana, Nigeria, Pharmacoepidemiology, (PubMed MESH terms)

## Abstract

**Background:**

Non-communicable diseases are a growing burden in many African countries; cardiovascular disease is the main disease. Antihypertensive medicines (AHM) are a common treatment option but we know little about community use in most low- and medium-income countries (LMIC). We aimed to describe the use of antihypertensive medicines (AHM) in Ghana and Nigeria using a novel data source.

**Methods:**

We used data from mPharma—a health and pharmaceutical company which distributes pharmaceuticals to hospital and retail pharmacies. We extracted data using the anatomical therapeutic chemical (ATC) classification codes and calculated use in defined daily doses and explored patterns by class, medicines, dose, and originator or generic product.

**Results:**

AHM use differed between Ghana and Nigeria. The most used classes in Ghana were angiotensin receptor blockers (ARB) followed by calcium channel blockers (CCB) and angiotensin-converting-enzyme inhibitors (ACEi). The five most used products were 16 mg candesartan, 30 mg nifedipine, 10 mg lisinopril, 5 mg amlodipine and 50 mg losartan. In Nigeria ARB, CCB and diuretics were widely used; the top five products were 50 mg losartan, 10 mg lisinopril, 30 mg nifedipine, 40 mg furosemide, and 5 mg amlodipine. More originator products were used in Ghana than Nigeria.

**Conclusion:**

The differences between Ghana and Nigeria may result from a combination of medical, contextual and policy evidence and reflect factors related to clinical guidance (e.g. standard treatment guidelines), accessibility to prescribers and the role of community pharmacies, and structure of the health system and universal health coverage including funding for medicines. We show the feasibility of using novel data sources to gain insights on medicines use in the community.

## Background

Non-communicable diseases (NCD) are the leading public health challenges globally in the twenty-first century resulting in ill health and death, economic loss, diminished quality of life, and poor social development in both high- and low-resourced countries [1, 2]. In the latter, NCDs are growing swiftly; 41 million people die each year (i.e. 71% of all deaths globally). Each year, more than 15 million people aged between 30 and 69 die from a NCD where 85% of these so-called ‘premature’ deaths occur in low-resourced countries [3]. Four NCDs account for most (> 80%) premature NCD deaths: cardiovascular diseases (CVD 17·9 million deaths annually) is double that of the next group with most deaths i.e. cancers (9·0 million) with substantial burdens from respiratory diseases (3·9 million), and diabetes (1·6 million) [4].

Hypertension—untreated or uncontrolled—is the single largest contributor to CVD causing stroke, heart failure, and coronary artery disease; it is also a major contributor to kidney disease [5].

Most guidelines recommend that hypertension is diagnosed when a person's systolic blood pressure (SBP) in the office or clinic is ≥ 140 mm Hg and or their diastolic blood pressure (DBP) is ≥ 90 mm Hg following repeated examination [6].

Africa represents almost half of all people with high blood pressure in the world (46%) exceeding the proportion of deaths in low-resourced countries (40%) [7]. The overall prevalence of hypertension in Nigeria ranges from 8 to 46% depending on the study target population, type of measurement, and threshold value for defining hypertension [8, 9]. The prevalence is similar in men and women (7.9–50.2% vs. 3.5–68.8%, respectively) and in the urban (8.1–42.0%) and rural setting (13.5–46.4%). The pooled prevalence increased from 8.6% in the only study in the 1970s (1970–1979) to 22.5% (2000–2011) [9]. It also varies across the geo-political zones [10]. In Ghana the pooled prevalence of hypertension from a recent meta-analysis was 27.0% (95% CI 24.0–30.0%); it was double in the southern coastal (28%, 95% CI 24.0–31.0%) and middle geo-ecological areas (29%, 95% CI 25.0–33.0%) compared to the northern areas (13%, 95% CI 7.0–21.0%) [11].

For countries with constrained health budgets, every effort needs to be made to reduce patients’ reliance on costly medical treatments, including for NCDs, for which medicines are often the mainstay of treatment to reduce associated morbidity and premature mortality [12–14] The two main treatment approaches for hypertension are lifestyle modifications (more fruits and vegetables, less fatty food, less salt, more exercise exercise) and antihypertensive medicines (AHM) comprising several therapeutic groups.

There are several studies of hypertension in Ghana [15] and Nigeria but few on the use of AHM; most are confined to hospital audits [16], with one  considering community use [17]. We aimed to describe the community use of antihypertensive medicines in Ghana and Nigeria using a novel data source.

## Methods

### Data sources

We used data from mPharma (https://mpharma.com/)—a health and pharmaceutical company based in Accra, Ghana. They distribute pharmaceuticals to hospitals and retail pharmacies and have aggregated data on distributed medicines. This study was granted exemption from ethics review by the University of Queensland, Australia (Ref no 2020000453, 4 March 2020). The research uses only existing collections of data that contain only non-identifiable data and is of negligible risk. The parties (SH, University of Queensland) and mPharma (ET) signed a non-disclosure agreement due to the commercial nature of the data. Only mPharma employees had access to the raw data, while SH analysed the de-identifiable data extracted at aggregated level.

### Data analysis

The data were extracted based on the Anatomical Therapeutic Chemical (ATC) classification codes [18] of antihypertensive medicines. These included antihypertensives (C02), diuretics (C03), peripheral vasodilators (C04), vasoprotectives (C05) beta blocking agents (C07), calcium channel blockers (C08) and agents acting on the renin-angiotensin system (C09). Each product was noted for its medicine class (e.g. a calcium channel blockers) medicine (e.g. amlodipine) and dose formulation (e.g. 5 mg tablets). The medicine classes included: centrally-acting adrenergic agent (AAC); peripherally-acting adrenergic agent (AAP); alpha adrenergic blocker (AB); angiotensin converting enzyme inhibitor (ACEi); angiotensin receptor blocker (ARB); beta blocker (BB); calcium channel blocker (CCB); diuretic (DU); and statin (ST in combination products). We excluded the use of hydralazine because it is not used as a first line medicine and only used for hypertensive emergencies where labetalol is contraindicated. We extracted data on sales volume for each month between 1 January 2016 to 31 October 2020.

To standardise the amount of drug dispensed, we used the defined daily dose (DDD) metric from the WHO Collaborating Centre for Drug Statistics Methodology [18]. This DDD is the average daily maintenance dose for a typical 70 kg adult when used for its main indication. The DDD is useful for comparing medicines use across patient populations and accounts for dose formulation (e.g. tablets and capsules) and quantity within a regular pack (e.g. 28 tablets). We calculated the DDD use using a modified formula: [N (dispensed prescriptions) × M (mass of dose) × Q (quantity of pack size)]/DDD.

We examined the data by country (Ghana and Nigeria), medicine class (e.g. calcium channel blockers, CCB), medicine (e.g. nifedipine), dose (e.g. 5 mg), and product type (originator brand or generic). We descriptively analysed the data using Microsoft Office Excel for Office 365.

## Results

### Use by class

AHM use differed between Ghana and Nigeria. The most used single-medicine classes in Ghana were ARB (39.2%) followed by CCB (32.1%), and ACEi (13.4%, Table 1). There was low use of diuretics (7.5%) and BB (6.7%). In Nigeria, however, the most used classes were ARB (26.7%), CCB (22.8%), diuretics (18.5%) and ACEi (14.6%, Table 1). In Ghana, single-medicine products were preferred (99.4% of total) than Nigeria (92.3% of total). Three single-class groups accounted for 84.7% of all use in Ghana (ARB, CCB and ACEi) and 68.0% of all use in Nigeria (ARB, CCB, DU).

**Table 1:** Use of antihypertensive medicines by class (defined daily dose [DDD] and proportion [%]) for single and combination products in Ghana and Nigeria

	Ghana		Nigeria	
	Use (DDD)	%	Use (DDD)	%
*Single*				
ARB	380,029	39.2	940,546	26.7
CCB	310,645	32.1	803,845	22.8
ACEi	129,783	13.4	514,422	14.6
DU	72,272	7.5	651,408	18.5
BB	65,393	6.7	211,322	6.0
AAC	4,528	0.5	107,321	3.0
AAP	–	0	23,466	0.7
Total	962,650	99.4	3,252,329	92.3
*Combination*				
DU + DU		0	218,619	6.2
BB + DU	342	0.0	10,421	0.3
CCB + ARB		0	12,360	0.4
CCB + ARB + DU		0	27,665	1.3
CCB + ST	5813	0.6	1290	0.0
ACEi + ARB			2	0.0
Total	6155	0.6	270,357	7.7

*ARB* Angiotensin receptor blockers, *CCB* calcium channel blocker, *ACEI* angiotensin-converting enzyme inhibitor, *BB* beta-blocker, *DU* Diuretic, AAP, AAC and ST are not conventional abbreviations. AAC will involve centrally acting drugs like methyldopa

The use of combination-class products was over tenfold higher in Nigeria (7.7%) than Ghana (0.6%, Table 1). There were only two combination classes used in Ghana: a CCB plus a statin (94.4% of all combination use) and BB plus diuretic (remaining 5.6% of all combination use). In Nigeria, combination products accounted for 7.7% of all class products used; one class group—a double diuretics combination i.e. amiloride plus hydrochlorothiazide—accounted for four fifths (80.9%) of all combination use. There was some use of a CCB + ARB + diuretic (10.2% of combination use; amlodipine plus valsartan plus hydrochlorothiazide), CCB + ARB (4.6 of all combination use; amlodipine plus telmisartan), and BB plus a diuretic (3.9% of all combination use; atenolol plus chlorthalidone, Table 1). The least used combination products included a CCB + statin (amlodipine plus atorvastatin), and ACEi + ARB (ramipril + felodipine, Table 1).

### Use by medicine

The five most used medicines in Ghana were candesartan, nifedipine, lisinopril, amlodipine, and losartan accounting for 79% of the top ten medicines (Table 2). The five most used medicines in Nigeria were losartan, lisinopril, nifedipine, furosemide, and amlodipine accounting for 74% of the top ten medicines (Table 2).

**Table 2:** Use of antihypertensive medicines by medicines (defined daily dose [DDD] and proportion within class [%]) for single products in Ghana and Nigeria plus top ten ranking and proportion

Class	Medicine	Ghana				Nigeria			
		Use (DDD)	% within class	Rank	% of top 10	Use (DDD)	% within class	Rank	% of top 10
ARB	Candesartan	260,561	68.6%	1	27.7	33,203	3.5%		
	Losartan	92,384	24.3%	5	9.8	517,910	55.1%	1	17.8
	Valsartan	27,084	7.1%	9	2.9	239,830	25.5%	6	8.3
	Telmisartan	–	0			147,807	15.7%	8	5.1
	Irbesartan	–	0			1796	0.2%		
	Total	380,029				940,546			
CCB	Nifedipine	160,490	51.7%	2	17.1	408,500	50.8%	3	14.1
	Amlodipine	101,778	32.8%	4	10.8	394,731	49.1%	5	13.6
	Felodipine	48,377	15.6%	7	5.1	600	0.1%		
	Nimodipine	–	0			15	0.0%		
	Total	310,645				803,845			
ACEi	Lisinopril	129,004	99.4%	3	13.7	418,073	81.3%	2	14.4
	Ramipril	779	0.6%			88,831	17.3%		
	Perindopril	–	0.0%			7170	1.4%		
	Captopril	–	0.0%			178	0.0%		
	Enalapril	–	0.0%			170	0.0%		
	Total	129,783				514,422			
DU	Indapamide	65,332	90.4%	6	6.9	74,382	11.4%		
	Furosemide	1968	2.7%			398,932	61.2%	4	13.7
	Hydrochlorothiazide	–	0.0%			148,244	22.8%	7^a^	5.1
	Bendroflumethiazide	4972	6.9%			21,731	3.3%		
	Metolazone	–	0.0%			5202	0.8%		
	Torasemide	–	0.0%			2917	0.4%		
	Total	72,272				651,408			
BB	Atenolol	26,810	41.0%	10	2.8	121,556	57.5%	9	4.2
	Carvedilol	29,275	44.8%	8	3.0	22,484	10.6%	6	8.7
	Metoprolol	7911	12.1%			40,816	19.3%		
	Propanolol	21	0.0%			11,274	5.3%		
	Bisoprolol	1377	2.1%			7016	3.3%		
	Labetalol	–	0.0%			8177	3.9%		
Total		65,393				211,322			
AAC	Methyldopa	4528	100			107,321	100	10	3.7

*ARB* Angiotensin receptor blockers, *CCB* calcium channel blocker, *ACEI* angiotensin-converting enzyme inhibitor, *BB* beta-blocker, *DU* Diuretic, AAP, AAC and ST are not conventional abbreviations. AAC will involve centrally acting drugs like methyldopa

^a^Amiloride + HCT was ranked #7 but removed as it is a combination product

The most widely used ARB in Ghana was candesartan (68.6 of all ARB) followed by losartan (24.3% of all ARB). Conversely, in Nigeria, losartan accounted for more than half of use (55.1% of all ARB) followed by valsartan (25.5% of all ARB) and telmisartan (15.7% of all ARB). Of the CCB used in Ghana, nifedipine was the most widely used (51.7% of all CCB) followed by amlodipine (32.8%) and felodipine (15.6%, Table 2). In Nigeria two CCBs were used almost exclusively: nifedipine (50.8% of all CCB) and amlodipine (49.1%, Table 2). In both Ghana and Nigeria the most commonly-used ACEi was lisinopril (99.4% of all ACEi in Ghana, 81.3% in Nigeria) and ramipril (0.6% of all ACEi in Ghana, 17.3% in Nigeria, Table 2).

The use of diuretics was substantially different across the two countries. The preferred medicines in Ghana were indapamide (90.4% of all DU), bendroflumethiazide (6.9% of all DU) and furosemide (2.7% of all DU, Table 2) whereas in Nigeria, the preferred DUs were furosemide (61.2% of all DU), hydrochlorothiazide (22.8% of all DU), and indapamide (11.4% of all DU, Table 2). In Ghana, three medicines accounted for most BB use: carvedilol (44.8% of all BB), atenolol (41.0% of all BB); and metoprolol (12.1% of all BB) but the pattern was different in Nigeria where the most widely used BBs were atenolol (57.5% of all BB), metoprolol (19.3% of all BB), and carvedilol (10.6% of all BB, Table 2).

### Use by dose formulation

In Ghana, the five most used single-medicine dose products were candesartan 16 mg (16.0% of all single product use), nifedipine 30 mg (12.4%), amlodipine 5 mg (10.6%, only dose used), candesartan 8 mg (8.7%), and losartan 50 mg (7.8%, Table 3). The top ten medicine dose products constituted 80.5% of all single medicine products used. In Nigeria, the five most used single-medicine dose products were furosemide 40 mg (12.2% of all single product use), amlodipine 5 mg (12.1%, only dose used), losartan 50 mg (8.3%), nifedipine 30 mg (8.2%), and lisinopril 10 mg (7.2%, Table 3). The top ten medicine dose products constituted 71.6% of all single medicine products used.

**Table 3:** Use of antihypertensive medicines by dose product: use in defined daily dose [DDD] and proportion within all single-medicine dose products [%] in Ghana and Nigeria

No	Ghana				Nigeria			
	Medicine	Dose (mg)	Use (DDD)	% all use	Medicine	Dose (mg)	Use (DDD)	% all use
1	Candesartan	16	153,812	16	Furosemide	40	397,284	12.2
2	Nifedipine	30	119,532	12.4	Amlodipine	5	394,230	12.1
3	Amlodipine	5	101,778	10.6	Losartan	50	271,533	8.3
4	Candesartan	8	83,653	8.7	Nifedipine	30	265,585	8.2
5	Losartan	50	75,340	7.8	Lisinopril	10	234,870	7.2
6	Lisinopril	10	72,950	7.6	Losartan	100	194,982	6
7	Indapamide	1.5	65,332	6.8	Lisinopril	20	149,234	4.6
8	Nifedipine	20	38,309	4	HCT	50	148,244	4.6
9	Lisinopril	20	37,944	3.9	Nifedipine	20	142,915	4.4
10	Atenolol	50	26,014	2.7	Valsartan	160	128,352	3.9
11	Candesartan	32	23,096	2.4	Valsartan	80	111,478	3.4
12	Lisinopril	5	18,110	1.9	Methyldopa	250	107,321	3.3
13	Valsartan	160	17,784	1.8	Telmisartan	80	92,180	2.8
14	Losartan	100	17,044	1.8	Indapamide	1.5	74,382	2.3
15	Valsartan	80	7720	0.8	Atenolol	50	73,747	2.3
16	Nifedipine	10	2648	0.3	Telmisartan	40	55,627	1.7
17	Methyldopa	500	2345	0.2	Losartan	25	51,395	1.6
18	Methyldopa	250	2184	0.2	Lisinopril	5	33,969	1
19	Furosemide	40	1951	0.2	Atenolol	100	31,380	1
20	Valsartan	320	1580	0.2	Candesartan	16	21,002	0.6
21	Atenolol	100	795	0.1	Atenolol	25	16,429	0.5
22	Furosemide	20	17	0	Candesartan	8	12,201	0.4
23	Atenolol	25	1	0	Furosemide	20	1648	0.1
24	Losartan	25	–	0	Amlodipine	2.5	501	0
25	Amlodipine	2.5	–	0	Valsartan	320	–	0
26	HCT	50	–	0	Nifedipine	10	–	0
27	Telmisartan	80	–	0	Methyldopa	500	–	0
28	Telmisartan	40	–	0	Candesartan	32	–	0
Total			962,650				3,252,329	
	Top 10 (% all use)			80.5				71.6

*HCT* hydrochlorothiazide

### Use by product type

The use of generic products (across all single and combination class products) dominated in Nigeria (78.4%) but they were less often used in Ghana (21.6%; data not shown).

The use of generic products for the five main classes of single products dominated in Nigeria (average 94.3%) but they were less often used in Ghana (average 30.8%). The proportional use of generic ARBs was 90.3% in Nigeria but only 24.5% in Ghana—a difference of almost four-fold; the proportional use of diuretics in Nigeria (88.1%) was nine times that of Ghana (9.6%, Table 4). The use of CCB in Nigeria was almost exclusively generic (97.9%) but lower in Ghana (59.4%). In four classes—BB, ARB, ACEI, and diuretics—the use of originator brands prevailed in Ghana whereas the use of generic products dominated in all five classes in Nigeria (Table 4).

**Table 4:** Use of antihypertensive medicines for the five main classes (defined daily dose [DDD]) and product type—generic or originator (proportion within class that is generic [%])—for single products in Ghana and Nigeria and combined countries plus difference between Nigeria and Ghana

Class	Type	Ghana		Nigeria		Nigeria/Ghana
		Use (DDD)	% generic	Use (DDD)	% generic	% generic
ARB	Generic	92,971	24.5	849,568	90.3	3.7
	Originator	287,058		90,978		
	Total	380,029		940,546		
CCB	Generic	184,622	59.4	787,031	97.9	1.6
	Originator	126,023		16,814		
	Total	310,645		803,845		
ACEi	Generic	24,534	18.9	501,055	97.4	5.2
	Originator	105,249		13,367		
	Total	129,783		514,422		
DU	Generic	6940	9.6	574,109	88.1	9.2
	Originator	65,332		77,299		
	Total	72,272		651,408		
BB	Generic	27,122	41.5	206,229	97.6	2.4
	Originator	38,271		5093		
	Total	65,393		211,322		

*ARB* Angiotensin receptor blockers, *CCB* calcium channel blocker, *ACEI* angiotensin-converting enzyme inhibitor, *BB* beta-blocker, *DU* Diuretic

## Discussion

Dispensed use of antihypertensives varied between Ghana and Nigeria. The most used medicine class in Ghana was ARB followed by CCB and ACEi. The five most used medicines in Ghana were candesartan, nifedipine, lisinopril, amlodipine, and losartan accounting for 79% of the top ten medicines. The five most used products in Ghana were candesartan 16 mg, nifedipine 30 mg, amlodipine 5 mg, candesartan 8 mg, and losartan 50 mg. The most used medicine class in Nigeria was ARB followed by CCB, then diuretics. The five most used medicines were losartan, lisinopril, nifedipine, furosemide, and amlodipine accounting for 74% of the top ten medicines. In Nigeria the top five products were furosemide 40 mg, amlodipine 5 mg, losartan 50 mg, nifedipine 30 mg, and lisinopril 10 mg. More generic products were used in Nigeria than Ghana. AHM use was broadly similar for both countries—ARB and CCB were the first and second most used medicines, but the third for Ghana was ACEi while for Nigeria it was diuretics. This difference may be attributed to the high cost of ACEi in Nigeria which makes it more difficult to afford, despite its proven advantages in terms of lowering blood pressure over diuretics [19].

A recent economic evaluation and budget impact analysis on the main antihypertensive therapeutic groups used for uncomplicated essential hypertension in a Ghanaian population [20] found that diuretics were more cost-effective than ACEi, ARB, or BB for first-line management of essential hypertension; this result was driven by the greater reduction in stroke incidence with diuretics. CCB were more effective than diuretics but were associated with higher costs. Nevertheless, both thiazide diuretics and CCB were regarded as cost-effective treatment options in Ghana, consistent with evidence also available from Nigeria [21]. Notably the 2017 Ghanaian Standard Treatment Guidelines stipulate a preference for TZDs and CCBs, and recommends against the use of ACEi as first line drugs for uncomplicated hypertension in black patients [22]. Despite these recommendations, we found that the most used medicine class in Ghana was ARB followed by CCB and ACEi. Data from the NHIS showed that CCB, followed by diuretics, are the most predominantly used in public health facilities in Ghana [23]. When choosing antihypertensive drug treatment for adults of black African or African-Caribbean family origin, the National Institute of Health and Care Excellence (NICE) in the UK says to consider an ARB, in preference to an ACE inhibitor [24].

The discordance between guidelines and practice may reflect, in part, that guidelines can be slow to influence practice if not supported by an active implementation strategy. The previous Ghanaian guidelines (2010) did not indicate any clinically-informed medicine preferences for managing uncomplicated essential hypertension, leaving the choice of first-line treatment open for the five main classes of AHM. In addition, the Ghanaian NHIS did not provide incentives to encourage the use of lower-priced formulations or better-value AHM classes. This appears to be an ongoing issue, given the relatively low levels of generic AHM use revealed in our study. The higher level of use of originator products may reflect prescriber and or patient preferences for these products perhaps linked to delayed NHIS reimbursement to providers, thus encouraging out-of-pocket expenditure, or related to concerns about quality [25]. In addition, it may also highlight the need for strengthened reimbursement and procurement policies—informed by health technology assessment (HTA)—to help ensure medicine costs better reflect their ‘value-based’ price. In that regard the recently launched Ghana Strategy for Health Technology Assessment (2020) sees HTA as a strategic instrument to inform the selection, pricing and procurement of pharmaceuticals as well as other health technologies, at least for the publicly -funded sector [26].

The use of combination products was tenfold lower in Ghana than Nigeria. Clinically, combination treatment may be preferred when existing monotherapy is not achieving adequate control and there is a reluctance to increase doses because of the risk of side effects [27]. It is not clear why the use of combination products were higher in Nigeria but some factors would likely be the availability of such products, out-of-pocket costs, patient and prescriber preferences, and the local epidemiology of hypertension. This warrants further investigation.

This is one of the first studies that we know of to report on the use of AHM at the community level in Ghana and Nigeria using a novel data source. We acknowledge three main limitations of our approach. Firstly, the data are extracted from a commercial source, which was not designed for research purposes so there may be some elements that are not available for analysis. Secondly, we do not have an adequate population denominator to ascertain absolute quantities of medicines use as per the DDD metric [18]. Thirdly, these are aggregated data and we cannot ascertain the indications for use as these AHM can be used to treat several CVD conditions nor the likely concomitant use of single AHM products at the same time.

Studies of AHM use in Nigeria have been inconsistent; most studies showed that CCBs, diuretics and BB are the most used AHM [28–31] but one study showed a preference for ACEIs, either as single or combination products [32]. The former three studies were based in single hospitals with few participants, whereas the latter study [32] was community-based with a large sample size that likely captures the AHM use irrespective of where they received their prescriptions (e.g. hospital, clinic). The use of particular AHM classes may be attributed to cost, prescriber’s preference, a reluctance to change medication, and perceived advantages of each class.

The use of combination AHM products is more common in Nigeria than Ghana. This observation is supported by other studies that show Nigerians prefer combination therapy; most study participants used combination therapy but only less than 20% [28], 5% [29] and 13% [32] of the study participants used monotherapy. The concomitant use of single AHM products can reduce the burden of adverse reactions of a single AHM used in high doses, in reducing cases of drug resistance, and in gaining better blood pressure control, although the cost might be higher and adherence more difficult. It also conforms to recommendations from the American Heart Association^23^ and to the recently revised Nigerian National Standard Treatment Guidelines [33]. Recent Nigerian studies have shown low adherence to concomitant use of single AHM products (1% [34], 4.1% [35], 8.9% [36], and 31.8% [37]) and this is consistent with a study that compared AHM adherence in Nigeria and Ghana [38]. On the contrary, adherence to AHM was found to be high in a Ghanaian study (89.2%, but not stated if fixed dose combination or concomitant single products) [39] and as reported by stakeholders in a qualitative study [40]. We emphasise the need for tailored adherence education and counselling for patients using concomitant AHM.

Nigerians use more generic products in the community whereas Ghanaians prefer originator products; consistent with other studies [41–43]. In Nigeria, the guidelines [22] and the essential medicine lists [44] contain only generic names, as does the Ghanaian NHIS medicine list [45]. In both Ghana and Nigeria, originator products are more expensive than generic products as they are imported [46]. Furthermore, there may be a wide range of costs for generic products in low and middle income countries (LMIC) which may be even more expensive than in higher income countries. People in LMIC disproportionately buy expensive branded generic medicines rather than cheaper unbranded medicines [47]. Although there are good reasons to support the local production of generic (i.e. off-patent) medicines [48], some prescribers and patients might trust originator products more [49]. The use of generic products reduced expenditure and out-of-pocket payments by hypertensive patients without affecting medicine utilisation [50]. This might also explain the dominance of generic use in Nigeria due to patient preference: the out-of-pocket payment for healthcare was 71.5% in Nigeria (based on national health accounts [51]) but only 40% in Ghana [52]. The AHM use in Ghana is broadly consistent with two other recent studies from Ghana using claims data from the NHIS (where CCB were the most widely used, preferred additional treatment was a diuretic) [23] and private health insurance companies [53].

Both countries adhered to the guidelines for the treatment of hypertension; they are using CCB, diuretics, ACEI, ARB, and BB medicines. The Ghana STG are not directive on class; any of the five classes of major antihypertensive drugs can be used as first-line treatment [22]. The Nigerian STG on the other hand stipulates the class of the first line medicines and the subsequent lines of medicines to be used [33]. The differences in selecting a particular AHM from the various classes may be largely due to prescriber preference and availability. The patterns of AHM use in Ghana are not consistent with the economic evaluation of AHM showing that DU are more cost-effective than CCB [20]. HTA can help to reduce costs within national or jurisdiction-based health insurance systems within the context of universal health coverage [54, 55].

We have revealed some interesting differences in AHM use in Ghana and Nigeria over five years. In future, it would be beneficial to examine AHM use over time to better understand how guidelines, for example, may influence prescriber preferences and medicines within a class. The economic evaluation of AHM in Ghana [20] could be replicated in Nigeria to better inform medicine use. We could also use data on medicines use to calculate potential savings (and overspend) given the patterns of AHM and the cost-effectiveness of particular AHM classes. Patient level data (e.g. from mPharma and NHIS) can provide powerful insights into the rational—or otherwise—use of medicines.

## Conclusions

The differences between AHM use in Ghana and Nigeria may result from a combination of medical, contextual and policy evidence [56–58] and reflect factors related to clinical guidance (e.g. standard treatment guidelines), accessibility to prescribers and the role of community pharmacies, and structure of the health system and universal health coverage including public funding for medicines. We show the feasibility of using novel data sources to gain insights on medicines use in the community.

## Data Availability

The data that support the findings of this study are available from mPharma but restrictions apply to the availability of these data, which were used under license for the current study, and so are not publicly available. Data are however available from the corresponding author upon reasonable request and with permission of mPharma.
